# Basic Processes in Dynamic Decision Making: How Experimental Findings About Risk, Uncertainty, and Emotion Can Contribute to Police Decision Making

**DOI:** 10.3389/fpsyg.2019.02140

**Published:** 2019-09-20

**Authors:** Jason L. Harman, Don Zhang, Steven G. Greening

**Affiliations:** Department of Psychology, Louisiana State University, Baton Rouge, LA, United States

**Keywords:** police decision making, decisions from experience, dynamic decision making, fear conditioning, first person shooter

## Abstract

In this paper, we review basic findings from experimental studies in judgment and decision making that could contribute to designing policies and trainings to enhance police decision making. Traditional judgment and decision-making research has focused on simple choices between hypothetical gambles, which has been criticized for its lack of generalizability to real world contexts. Over the past 15 years, researchers have focused on understanding the dynamic processes in decision making. This recent focus has allowed for the possibility of more generalizable applications of basic decision science to social issues. We review recent work in three dynamic decision-making topics: dynamic accumulation of evidence in the decision to shoot or not shoot, how previous decisions influence current choices, and how the cognitive and neurological processing of fear influences decisions and decision errors. We conclude this review with a summary of how basic experimental research can apply in policing and training.

## Introduction

Police decision making is dynamic. Decisions unfold over time with information (often incomplete) becoming known at different rates. The same type of decision may be made multiple times, allowing for a police officer to base a future decision on past outcomes. And decisions are made in uncertain and often changing environments. While the early pioneers of judgment and decision-making (JDM) research highlighted the importance of uncertainty and changing dynamics in decision making (i.e., Simon, 1955; Edwards, 1961), this focus was largely ignored for many years in favor of studying simple one-time decisions which could easily be represented in controlled experiments using choices between monetary gambles such as choose between a sure gain of $5 or a 50/50 chance of getting $11 or nothing (i.e., Lichtenstein and Slovic, 1971; Kahneman and Tversky, 1979). One reaction to this focus on simple decision contexts was the field of *naturalistic decision making* (NDM) which focuses on describing and understanding how people make decisions in real contexts with real world constraints (Lipshitz et al., 2001). NDM typically focuses on highly specialized experts in high stake decision circumstances, such as firefighters, military personnel, and police. This extremely successful tradition of research has grown largely from the work of Klein (1989, 1993, 1997) and is often seen in contrast to traditional JDM research programs (more concerned with the experimental control of laboratory studies) and has had more relevance to topics such as police decision making than traditional JDM research (e.g., Klein et al., 2014). In this article, we review recent experimental work in JDM that has brought dynamic decision making back into focus and discuss some of the experimental findings from this work that could contribute to improving police decision making. We begin by reviewing recent research on the decision to shoot or not shoot which analyzes the decision to shoot or not as a dynamic accumulation of evidence over time. We then review recent research on repeated experiential decisions and how they differ from one-time decisions typically studied in the lab. Finally, we review the neurocognitive mechanisms of fear in decision making. The primary goal of this article is to review recent experimental JDM research that has more fidelity with applied settings relevant to police decision making. A secondary goal of this article is to prompt future research incorporating findings from dynamic decision making to applied settings in a police context.

## Dynamic Accumulation of Evidence in the Decision to Shoot or Not Shoot

**Figure 1:**
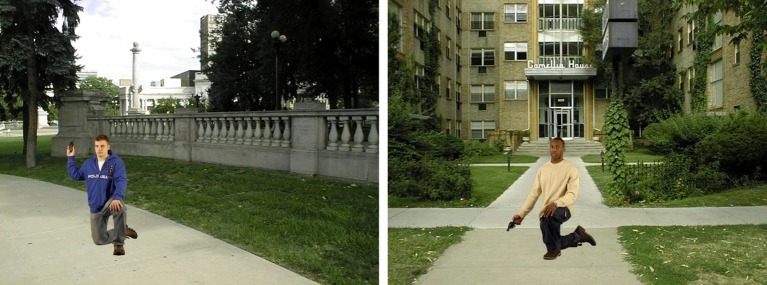
Example stimuli of the first-person shooter task (http://psych.colorado.edu/~jclab/FPST.html).

The experimental paradigm most closely analogous to a consequential decision made by police officers is likely the decision to shoot or not shoot. Shooting decisions have been researched extensively in a simulated video game task (Correll et al., 2002, 2006). In this task, participants are presented with a series of background and target images (e.g., Figure 1). Each image contains a target holding various objects. In the task, participants are usually instructed to decide – as quickly as possible – whether the target is holding a gun. If a gun is identified, the participant is instructed to shoot as quickly as possible by pressing a button on the computer. In the context of police shooting decisions, the target may vary in sex, race, and appearance.

A large body of research has relied on this paradigm to examine factors (situational, individual, and contextual) of both weapon identification and shoot (vs. do not shoot) decisions. A majority of the early research focused on the shooting decision as a function of the decision maker and target’s race. Correll et al. (2002), for example, found that White participants made more correct decisions to shoot an armed target who was African American, than when the target was White. Participants were also more likely to correctly decide to “not shoot” an unarmed target who was White. Correll et al. (2007) found that police officers were on average more accurate than community participants in their shooting decisions, whereas community members made more errors by setting a lower decision threshold for shooting a Black target. Decisions to shoot, however, were influenced by a number of other factors such as the mode of the presentation (video vs. picture, Cox et al., 2014), training (Correll et al., 2007), and contextual cues (neighborhood safety) (Kahn and Davies, 2017).

Cognitive factors also play a role in the accuracy of shooting decisions. For example, decision makers under high working memory load were more likely to shoot an unarmed target and more likely to make errors (e.g., shooting an unarmed target) (Kleider and Parrott, 2009; Kleider et al., 2010). Relatedly, physical fatigue – as operationalized by sleep – also had a negative effect on shooting accuracy (Ma et al., 2012). Although a large body of work on police shooting decisions has focused on the difference between Black vs. White targets, some scholars have begun examining the role of gender, SES, and other racial and ethnic backgrounds (e.g., Latino, Asian, Muslim: Fleming et al., 2010; Plant et al., 2011; Sadler et al., 2012; Moore-Berg et al., 2017).

Arguably, the classic police shooting task, which relied on statistical analysis of correct and incorrect decisions, has overlooked important temporal dynamics of the decision-making process (Pleskac et al., 2018). Pleskac and colleagues noted that the typical approach assumes that “all the information used to make a decision is extracted from the scene in a single sample” (p. 1302). Under a dynamic model of decision making called the drift diffusion model (DDM), the decision to shoot (or not shoot) can be modeled as a dynamic process where the police officer accumulates momentary evidence over a short period of time and makes a rapid decision (Ratcliff and Rouder, 2000; Klauer and Voss, 2008). The DDM describes how decisions unfold over time as a function of accumulated evidence, and it can be used to predict both choice and response time. Based on the DDM, decision makers extract information from the decision context, which is accumulated as evidence for or against the decision to shoot.

By integrating the dynamic drift diffusion model to the classic first-person shooting task, Pleskac et al. (2018) found that the race of the target did not affect the prior bias of decision makers to shoot Black targets. However, when examining the specific decision processes under the dynamic drift diffusion model, the authors found a quicker rate of evidence accumulation when faced with a Black target. In other words, participants gathered evidence toward their decision quicker when the target was Black. Moreover, the authors found that some participants were more conservative in their decision, such that they set higher evidence thresholds when faced with a Black target. In other words, participants required more information before making a decision to shoot a Black target. Extending this work, Johnson et al. (2018) also found that untrained civilians gathered evidence quicker when faced with a Black target. This bias, however, was not observed in trained officers. The authors also found that providing decision makers with prior information – similar to police dispatch calls – eliminated racial bias at both the process and behavioral level.

The novel application of the dynamic drift diffusion model on the classic police shooting task affords future research to more finely examine how race, experience, and training combine to affect the decision-making process. Specifically, this research suggests that the various components of the decision process may be compensatory in producing the final decision. For example, a slower accumulation process and a low decision threshold may produce similar final decisions than a quick accumulation process but a more stringent decision threshold. Moreover, the presence of a decision bias may be attributed to different psychological processes (e.g., initial bias, rate of evidence accumulation, decision threshold). Taken together, under the dynamic drift diffusion model, it is possible to observe similar decision outcomes between different decision processes.

## Decisions From Experience

Traditional research in the field of human judgment and decision making relied on experimental paradigms where participants would make a choice between two options, usually monetary gambles where the outcomes and associated probabilities were presented (e.g., Kahneman and Tversky, 1979; Kahneman et al., 1982). For example:

Which of the following would you prefer?

**A:** a 0.8 chance to get $4 and 0.2 chance to get $0.

**B:** get $3 for sure.

This work was very influential and led to codified “knowledge” about how people react to the prospect of risks. One of the main findings from this line of work is that people overweigh or overreact to the potential of rare but serious outcomes. More recently, researchers have realized that these one-time choices with full information (referred to henceforth as decisions from description: DFD) do not accurately model how most of our decisions (and police decisions) are made. More typically, we face choices that we have made previously and will make again and we rarely have full knowledge of the exact risks associated with different possible outcomes. Likewise, typical police decisions such as whether to pull someone over are not analogous to the DFD example above. To address this, researches created new experimental paradigms where people make repeated decisions and learn about the likelihood or risks of different outcomes primarily through their experience (decisions from experience: DFE; Hertwig et al., 2004). For example, instead of choosing between options A and B above with their full description, participants would make repeated choices between two unmarked buttons, each time they chose one option they would receive $3 and when they choose the other button they would receive $4 80% of the time and $0 otherwise. Results from these new paradigms have found that choice behavior is quite different and sometimes in the opposite direction when made repeatedly with experience when compared to one-time choices with full information. These systematic and robust differences between decision behavior from experience and from description have been labeled *the description-experience gap* (Hertwig et al., 2004; Hertwig and Erev, 2009). This gap makes explicit the differences in decision behavior when outcome relevant information is acquired through experiencing sequential outcomes from choice options as opposed to from a descriptive summary of the choice options.

In context, underweighting rare events through experience could explain anomalies such as low levels of flood insurance in areas where flood insurance is advised (if not mandated). When someone decides to purchase flood insurance, he/she is protecting himself/herself from the possible devastating consequences of a flood. How much this rare possibility motivates decision makers depends on how they evaluate the risk. If they were making a one-time hypothetical choice with full information of the actual likelihood of a flood along with the potential costs, they would likely fall in line with traditional findings from DFD and react strongly to the risk of floods. If however, they had lived in the same location for a long time without experiencing a flood (or even experiencing a flood but very rarely), they would react less strongly to the same objective risks. Key to this example is a key finding by Larraharge and Gonzalez (replicated over several conditions by Erev et al., 2017) that when making repeated decisions while you are presented with descriptions of risk leads to behavior early in an experiment akin to DFD (i.e., overweighting of rare events), but after only a few rounds of feedback these descriptions are largely ignored and behavior is identical to typical DFE choices (underweighting of rare events). One important caveat to the DFE finding that rare events are underweighted was found by Harman and Gonzalez (2015). Their research which combined behavioral choice data and computational cognitive modeling found that reactions to rare negative outcomes change depending on the recency of a negative rare outcome but more importantly by how often a person has experienced a positive outcome in the same circumstance. In other words, a person’s likelihood of renewing flood insurance will vary depending on when the last flood was and more so, how long they have owned their house (with long time residence being more likely to cancel flood insurance).

As critical decisions made by police are typically faced repeatedly, we believe insights from DFE may be more informative than typical DFD research. Most notably, reactions to rare negative events are different and more complex than the traditional finding from DFD that people overweigh or overreact to rare consequential outcomes. Several DFE studies have demonstrated that people act as if they underweigh the probability of rare events compared to defined probability when making decisions based on repeated experienced outcomes (Hertwig et al., 2004). For example, when choosing between a safe option and a risky option with a high or low outcome, participants prefer the risky option when the high outcome is likely, but they prefer the safe option when the high outcome has a probability of around 0.2 or less. This pattern is reversed when the same options are presented with full written descriptions (Erev et al., 2010). The *description-experience gap* has been found to be a robust phenomenon (Barron and Erev, 2003; Hertwig et al., 2004; Gonzalez and Dutt, 2011) with the reversal in reactions to rare outcomes being the most consequential finding.

In terms of police decision making and use of force in particular, we believe that JDM’s recent focus on dynamic experiential decision making is an important step in bringing laboratory research closer to real world applications for police decision making and training. In terms of the focus of this special issue, we think of the use of lethal force as a reaction to a rare consequential event. A vast majority of encounters made by police officers result in a non-violent outcome on both sides. On very rare occasions, police find themselves in situations that may warrant the use of force while situations which warrant the use of lethal force are even more rare. Error rates in the use of lethal force then would represent the overweighting or overreaction to the actual risks in the environment. The results from Harman and Gonzalez (2015), that the overreaction to rare events is dynamic and depends on the frequency of non-consequential outcomes experienced, would predict that seasoned officers with years of experiencing encounters resolved without the use of force would be less likely to overreact to low likelihood risks than newer officers. This hypothesis however may only capture part of risk perceptions when making repeated decisions. As will be outlined in the next section, our perceptions of risks may not be influenced solely by actual outcomes experienced in the past, but also by the outcomes of others (through social communication or media coverage), imagined outcomes, expectations, or simulated outcomes (i.e., training). All of these taken together could easily create a decision context where the rare outcome that warrants the use of lethal force is perceived as not that rare at all. Therefore, simply educating officers of actual base rate risks would be ineffective. Rather, repeated experiences of positive outcomes – either imagined or in training, may be an effective way to moderate the weighting of rare events, thus reducing error rates. Thus, the literature would recommend creating more positive outcomes in training scenarios, rather than a central focus on lethal force outcomes.

## Neurocognitive Influence of Fear on Decision Making

Police as a group have significantly more exposure to traumatic events, such as being assaulted, viewing assault, or viewing a dead body, than those in the general population (Haugen et al., 2012; Federal Bureau of Investigation, 2017; Morgan and Kena, 2018), which appears associated with psychological distress and worry (Leino et al., 2011). They also appear to be more likely to experience post-traumatic stress disorder than the general population (Kessler and Chiu, 2005; Maia et al., 2007; Klimley et al., 2018) and may be at greater risk for death by homicide and suicide compared to the general population (Violanti, 2010). How might either having such experiences or even simply learning about them affect one’s emotional state and subsequently one’s decision making?

One likely important factor in police decisions involves the impact of emotions on decision making, particularly fear. For example, a state of fear has been associated with increased processing of aversive information (Robinson et al., 2011). On the other hand, a state of fear has also been found to improve response inhibition. Specifically, participants made fewer commission errors on no-go trials of a go/no-go task while under threat of shock versus safe (Robinson et al., 2013). One avenue for potentially bettering our understanding of how emotions affect decision making for police officers is to first consider the basic science of fear (or threat) conditioning.

One of the most common ways of studying fear (or the anticipation of threat) is fear conditioning *via* classical conditioning. In classical fear conditioning, a neutral stimulus such as an auditory tone or a visual object (i.e., the conditioned stimulus with reinforcement, CS+) is paired with an inherently aversive stimulus such as a mild shock or the sound of nails on slate (i.e., the unconditioned stimulus, US). Successful classical fear conditioning has occurred if the CS+ elicits a measurable conditioned response (CR), such a skin conductance response (SCR), similar to, though often of lesser magnitude than, the unconditioned response (UR) elicited by the US alone. Often, classical fear conditioning involves having participants differentiate the CS+ from a second neutral stimulus that is never paired with the US (i.e., the conditioned stimulus without reinforcement, CS−). In cases of differential fear conditioning, one will observe a significantly greater CR for the CS+ compared to the CS−. Based on recent meta-analytic evidence of brain imaging data (Fullana et al., 2016), a greater response to the CS+ versus the CS− is found in several brain areas, most notably bilateral anterior insula (aIn) and bilateral dorsal anterior cingulate cortex (dACC).

Relevant to the current topic, humans can acquire fear conditioning in two additional scenarios, neither of which involves the actual experience of the US. The first is vicarious fear conditioning (which has also been observed in non-human primates) in which participants develop a fear CR by observing another person undergo a classical fear conditioning experiment. In such cases, participant who never experienced the CS+ paired with the US nevertheless evinces a CR when viewing the CS+ versus CS−, such as a differential SCR (Olsson and Phelps, 2004) and differential activation of the aIn and dACC (Olsson et al., 2007). The second method by which humans can acquire fear associations is *via* instructed fear learning. In such experiments, participants are first told explicitly that one of the conditioned stimuli will be paired with the US and the other will never be paired with the US. Later, participants are shown both the CS+ and the CS−, though neither is ever physically paired with the US. As with both classical and vicarious fear conditioning, instructed fear conditioning has been observed as both a greater SCR and more activation in the aIn and dACC when viewing the CS+ compared to the CS− (Olsson and Phelps, 2004; Mechias et al., 2010). Taken together, to the extent that a fear response can affect one’s decision making at any one moment, not only are the fear-related experiences one has had important, but of potential concern are the fear-related experience has one seen or heard about.

Finally, when considering the potential role of fear in police decision making, one should also consider what might broadly be called context-dependent fear conditioning. This includes fear conditioning to environmental cues *per se* or the role of environmental cues as “occasion setters” (i.e., when a certain context qualifies the significance of a given CS) (Maren et al., 2013) and non-reinforced stimuli with semantic or conceptual connections to the CS+. Regarding the former, physical environments that are paired with the US elicit greater SCRs and activation of the aIn than environments never paired with shock (Alvarez et al., 2008; Marschner et al., 2008). Other research has found that environmental information modifies participants’ response to conditioned stimuli. When the environmental context predicts that a cue will be paired with the US, then participants have a larger CR, compared to when the environmental context predicts that the cue is not associated with receiving the US, though in both conditions, participants receive the same number of USs (Indovina et al., 2011). In the case of decisions to shoot, one recent study found that participants were more likely to make decisions to shoot in perceived threatening neighborhood compared to a perceived safe neighborhood (Kahn and Davies, 2017). Other contextual factors that appear to impact fear conditioning include semantic similarity and conceptual connections. For example, participants who are fear conditioned to a specific word display generalized fear conditioning (i.e., conditioning to a non-reinforced CS) to an orthographically distinct yet semantically related word (Boyle et al., 2016; Grégoire and Greening, 2019a). Additionally, conceptual factors such as category membership can lead to generalized fear conditioning. For example, pairing 50% of animal pictures in a set with shock produced a generalized CR to the other animal pictures in the set that were never paired with shock (Dunsmoor et al., 2012).

It is possible that police experience legitimate states of fear, while nevertheless overestimating the relative risk present in any one situation. Such fear can be acquired from experience, observation, or word-of-mouth, and can potentially affect decision making. On the other hand, however, civilians may underestimate the degree of legitimately fear-producing experiences that police draw upon, implicitly or explicitly, during any given situations. This may be because civilians have relatively few fear-related instances to draw upon when imagining how they would have reacted in a given situation. Regarding police, one potentially beneficial strategy might be to intervene in the attenuation of fear associations and the calibration of fear reactions to contextual factors.

One of the common treatments used in disorders associated with fear, such as PTSD, is imaginal exposure therapy (Holmes and Mathews, 2010), which has also been used for police officers (Haugen et al., 2012). To date, there is little experimental evidence detailing the most important factors and mechanisms by which imagery can be used to attenuate fear-conditioned associations, though this has recently begun to change. For example, either repeatedly imagining or viewing the CS+ without the US produced a similar degree of fear extinction (Reddan et al., 2018). Another way to attenuate fear is *via* reconsolidation. In fear reconsolidation, a memory probe of the CS+ is presented followed by a brief (10 min to 1 h) waiting period, followed by extinction trials. This procedure may produce more durable fear attenuation such that the fear-conditioned response is less likely to spontaneously recover (Schiller et al., 2010; Agren et al., 2012). Recent research has found that mental imagery can be used in fear reconsolidation procedures, for example by having participants simply imagine the memory probe to open the reconsolidation window (Grégoire and Greening, 2019b) or having participants imagine the CS+ during the extinction trials once the reconsolidation window is opened (Agren et al., 2017). Additionally, there are situations in which one might experience a fear response acutely. In such situations, active down-regulation of the fear response can be attempted using strategies of cognitive control or reappraisal. For example, Delgado et al. (2008) had participants down-regulate (i.e., suppress) their fear response to a CS+ by diverting their thoughts to “something calming in nature.” This attenuated both the SCR to the CS+ and brain activity in parts of fear conditioning network including the insula.

Police attend annual recertification training in which they undergo many varied “police call” simulations and interactions, either *via* video or live with individuals acting out a scene. Police officers are required to resolve the situation while their performance is being assessed. The results of the assessment either requalify the officers to handle their gun and go back out in the field, or they would not. While it may not be desirable for training to excessively suppress fear responses to threat, there could be a legitimate need to improve the accuracy of the fear response. Can this training help to improve the calibration of the fear-conditioned response? There is no clear answer to the question, but if one were to undertake in answering it, here are some factors one might want to consider. The goal of such training could aim to improve the discriminability of the fear response, with the aim that this improvement would also improve decisions to use force. In terms of the fear response, the goal could be to maintain “hits” of the fear response (i.e., fear when threat is present) while reducing “false alarms” of the fear response (i.e., fear when threat is not present). Furthermore, we may want to reduce “misses” of the fear response (i.e., no fear when threat is present) and maximize “correct rejections” of the fear response (i.e., no fear when threat is not present). In order to determine how police training affects the fear response, however, we need to consider the multiple factors that have been described above. In terms of decisions to use force, we might ask how the presence of a fear response affects the parameters involved in making a shoot/no-shoot decision.

The training of police officers might wish to employ a selection of scenarios that considers the calibration of the fear response. This might involve practicing proportionately fewer scenarios in which a threat is actually present. Additionally, there could be a need to facilitate positive learning such that it modifies the various contextual factors that can influence one’s fear response. Such positive experiences could involve aspects of community in addition to training simulations. For example, community engagement including both formal (e.g., organized social gatherings) and informal (e.g., day-to-day social encounters such as door-holding or gestures “good-morning”) positive encounters could help with the accurate calibration of the threat response by minimizing the influence of misleading contextual factors. Finally, such calibration through training and community engagement can never be perfectly achieved. In practice, officers can also practice threat regulation strategies such as cognitive reappraisal in potentially threatening situations.

## Conclusion

Though the criticism that traditional judgment and decision-making research (focused primarily on simple one-time choices between hypothetical gambles) lacks generalizability to consequential real world decision making has some validity, recent trends have focused on the dynamics of decision making which provide more fidelity with real world decision contexts. We reviewed recent findings that highlight dynamic pre-decisional processes that influence the type of consequential decisions police face in the line of duty. Work by Pleskac et al. (2018), applying drift diffusion modeling to decision to shoot or not, highlights how bias to shoot is not a singularly straightforward variable, but instead could influence how evidence in favor of a decision is accumulated or how much evidence is needed to make a decision. Likewise, the emerging topic of decisions from experience illustrates that reactions to risk and possible outcomes are strongly influenced by previous decisions made in similar contexts. And the review of the neurocognitive mechanisms of fear adds the insight that these previous experiences may be as subtle as imagined or vicarious scenarios.

While the focus on dynamic processes in decision making is an improvement in terms of generalizability, there is still a long way to go before laboratory-based decision-making researchers can offer concrete prescriptive suggestions for police training and operation. Any worthwhile intervention or policy should be theory driven, which the research above could assist in, but also needs to be empirically tested and validated. Some possibilities drawing from the research reviewed could be increased training of typical police scenarios that have positive outcomes as well as post incident interventions to moderate the neurological conditioning of fear responses with particular contexts.

## Author Contributions

JH organized the writing of this manuscript and wrote sections “Introduction,” “Decisions From Experience,” and “Conclusion.” DZ wrote section “Dynamic Accumulation of Evidence in the Decision to Shoot or Not Shoot.” SG wrote section “Neurocognitive Influence of Fear on Decision Making.”

### Conflict of Interest

The authors declare that the research was conducted in the absence of any commercial or financial relationships that could be construed as a potential conflict of interest.
